# Personal Need for Structure and Fractions in Mathematical Education

**DOI:** 10.3390/ejihpe12050033

**Published:** 2022-04-29

**Authors:** Valéria Švecová, Ľubomír Rybanský, Gabriela Pavlovičová

**Affiliations:** Department of Mathematics, Constantine the Philosopher University in Nitra, Tr. A Hlinku 1, 949 74 Nitra, Slovakia; lrybansky@ukf.sk (Ľ.R.); gpavlovicova@ukf.sk (G.P.)

**Keywords:** personal need for structure, mathematical knowledge, fraction test, Sfard’s theory of reification, the IRT model

## Abstract

The research was aimed at finding relations between mathematical knowledge and cognitive individual variable. We realized the experiment with 162 students of the Constantine the Philosopher University in Nitra, Slovakia. We had two variables—the personal need for structure (PNS) as a cognitive-individual variable and knowledge of the fraction as a mathematical variable. The relationships between the factors of the personal need for structure scale and the knowledge of fractions were determined by the IRT model. We have proven a negative correlation between the successful solving of fraction test and score in the PNS scale. This means that the higher the success rate of solving the fraction tasks, the lower the overall score on the personal need for structure scale and its subfactors.

## 1. Introduction

Mathematics is still one of the challenging subjects, despite the various innovative approaches in education. Mathematical formulas and algorithms can be considered structures. Therefore, we wondered whether there was a relationship between mathematical knowledge and the personal need for structure. Personal need of structure is defined as the need for the simple structure, transparency of things, and environment. This structure can be a kind of motive and desire, can manifest itself in the simplification of complex information, and can affect its cognitive processing. We can find individual differences in the personal need of the structure.

### 1.1. Previous Research

Categorical thinking is not inherently maladaptive; connecting past and present experiences enable swift decision making [1]. Persons high in the need for a simple structure have tendential to create broad connections to reduce confusion and assuage anxieties [2].

It was identified two conceptual different factors of the need for structure—the desire for structure (DFS—to have a structured environment) and response to the lack of structure (RLS—an individual’s response to the lack of structure in a specific situation) [2].

The desire for the structure factor: the extent to which the persons want to establish a structure in their daily lives. Individuals with a high desire for structure need a clear and structured way of life and a certain place for everything.

The response to the lack of structure factor: the extent to which the persons respond to unstructured, unpredictable situations. Individuals who expressively dislike uncertain situations or changes in their plans at the last moment achieve a high score in the response to the lack of structure.

Burns and Isbell [3] analyzed the need for simple structure and the success or failure of the processing of math information. They proved the dependence between mathematical intelligence and simplified categorical thinking. Advanced mathematical thinking today involves the use of cognitive structures. They are produced by mathematical activities to create new ideas based on the knowledge system and expand it. [4]. One current trend is investigating the development of cognitive structures for rational numbers. One of the biggest research projects was realized more than 30 years was the NSF-supported (National Science Foundation) Rational Number Project started in 1979. It was based on the work of [5,6,7,8]. The interest was in exploring the “zone of proximal development” of children’s rational-number concepts. Vygotsky described the schemas children typically use to process rational number information and interpreted rational-number situations. He also described how these schemas change because of theory-based instruction [9]. Several researchers emphasize the use of various image models and activities to properly build an understanding of the concept of fractions [5,10,11,12]. According to [13], the rational-number concepts are among the most complex and important mathematical ideas children encounter during their presecondary school years. We can see it from a variety of perspectives:

(a) The practical perspective: the ability to deal effectively with these concepts improves the ability to understand and handle situations and problems in the real world.
(b) The psychological perspective: rational numbers provide a rich environment for development and expansion of the mental structures necessary for continued intellectual development.
(c) The mathematical perspective: rational-number understandings create the foundation upon which elementary algebraic operations can later be based.

In our research, we are based on a psychological perspective in which fractions present a mental structure. Fractions, fractions’ task and their solutions and algorithms represent a certain structure in our research. We believe that this aspect could be related to students’ difficulties in solving fractional tasks.

### 1.2. Personal Need for Structure and Mathematics

More research deals with relations between PNS and mathematics, e.g., the impact of math anxiety and personal need for structure on the mathematical thinking [14]; the impact of personal need for structure on geometry—determination whether a high level of PNS is related to the lower performance on tasks in terms of understanding the construction of complex spatial structures [15]; the impact of PNS and the algorithms for operations with fractions which can be considered as structure [16]. Students can learn algorithms for addition, subtraction, multiplication, and division quickly. Therefore, they can correctly perform arithmetic operations with fractions. In this way, they gain the certainty that they understand the fraction correctly. Younger pupils prefer visual representation or trial-and-error methods, but older students prefer solving by algorithms, schemes, and symbolic representations [17].

Previous research showed relations between the need for structure and the success rate in mathematics. It was discovered an inversely significant dependence between the PNS and solving word problems with fractions. [16]

### 1.3. Research

We looked at the issue of fractions from several perspectives. The aims of our research were:

1. The application fraction test in Slovak language and Slovak conditions;
2. The analysis mistakes of students;
3. The relationship research between mathematical knowledge and cognitive individual variable. Mathematical knowledge is presented by knowledge of fractions, cognitive-individual variable is differentiated as personal need for structure (PNS).

## 2. Materials and Methods

The research sample consisted of 162 participants, students of first and second grades of Preschool and Elementary Education (PEP) and The Teacher Training for Primary Education (UPV) at the Constantine the Philosopher University in Nitra. Later, these students will be teachers for the primary level of elementary education, and mathematics is very important there. Data collection was conducted in 2016. The mean age of the PEP sample was 19.7 years (SD = 1.2) and the mean age of the UPV sample was 22.8 years (SD = 1.3). The study was carried out in accordance with the criteria of pedagogical research and approved by the Constantine the Philosopher University in Nitra.

In the first part of the research, participants completed a self-report of the PNS scale. After completing PNS, participants have solved the fraction test.

### 2.1. PNS Scale

The PNS scale by [18] is a six-point self-assessment scale. Participants evaluate statements focused on the structure of the environment. The scale contains 11 items and participants respond using a 6-point Likert scale. Although the original scale had 12 items, the authors followed the recommendation [2] and omitted item 5 due to its possible misinterpretation.

Several authors have investigated the internal structure of the PNS scale [2,18,19] by confirmatory factor analysis (CFA). Results of exploratory and confirmatory analyses show that a two-factor model fits the data better than a one-factor model. Were identified two factors *desire for structure* (DFS) and *reaction to lack of structure* (RLS). *PNS* is composed of two subscales consisting of the person’s desire to have a structured environment and the person’s response to lack of structure in each situation [20].

Items 3, 4, 6, and 10 belong to the DFS factor. Items 1, 2, 7, 8, 9 11, and 12 belong to the RLS factor.

### 2.2. Knowledge of Fractions as a Variable

Cognitive psychologists and educators have tried for several years to understand individual differences in knowledge and their impact on learning [21].

Through fractions, teachers can discover pupils’ understanding of numbers and relations among them. Pupils understand numbers not only through formal knowledge in a classroom but through personal experiences and intuitions. Rational numbers create important prerequisite conceptual foundations for the growth, understanding of other number types, and development of algebraic thinking [22].

To understand the ideas of rational numbers, one must have adequate experience with their many interpretations. The concept of the fraction is very comprehensive. It consists of several subconstructs. For building the correct concept of the fraction is necessary the understanding of each of these different meanings of fractions [5]. He described 7 different interpretations of rational numbers which contain indicators of the major mathematical structures and long-term mathematical goals, the related cognitive structure, and the related instructional structure. These interpretations consisted of rational numbers as fractions, as equivalence classes of fractions, as ratio numbers, as operators and mappings, as elements of a quotient field, as a measure, and as decimal fractions. They were based on three different ways in which mathematical concepts can be organized:

(a) Mathematical structures: a set of concepts is organized by mathematics;
(b) Educational structures: a set of concepts is organized by a teacher, textbook, or curriculum;
(c) Cognitive structures: a set of concepts is organized by a pupil.

We can find several pedagogical researchers who support almost any conceivable connection between these three types of structures (e.g., cognitive structures determine educational structures [23]; educational structures determine cognitive structures—[24]; disciplinary structures determine educational structures—[25].

First, four subconstructs of fractions were defined: measure, ratio, quotient, and operator [6]. Kieren avoided identifying the concept part-whole as a separate, fifth sub-construct, claiming that the term was included in all other sub-constructs. Only later the part-whole relationship was considered the fifth subconstruct of fractions [26]. According to several theories, pupils acquire new mathematical ideas procedurally or conceptually [4,27]. Procedural knowledge contains processes, algorithms, actions, and processes of actions [27,28]. Conceptual knowledge is the knowledge loaded in relationships [28]. Conceptual knowledge is a structural concept for which a mathematical concept is an abstract object/static structure [27]. In the process of concept formation, operational conceptions precede the structure. We can see mathematics knowledge as a kind of hierarchy, in which what is conceived purely operationally at one level should be conceived structurally at a higher level. Three steps in the process of concept formation were defined. These three stages correspond to three “degrees of structuralization”, which may be named on the grounds of purely theoretical analysis of the relationship between processes and objects. These three stages in concept development are interiorization, condensation, and reification:

Interiorization contents processes which lead to new knowledge at a lower level of mathematical ideas about subjects.

Condensation makes it easier to combine processes with other processes, compare and generalize. Students create their schemes and thought processes, they have some idea of the concept.

Reification—the ability to see something known completely differently. The student can examine the general characteristics of the categories and the various relationships between the various representatives [27].

According to Sfard’s theory of reification, the researchers Pantziara and Philippou proposed a test for the identification of the level of pupils’ conception of fractions (The full test is available at https://link.springer.com/article/10.1007/s10649-011-9338-x). The test consists of 21 tasks. They are divided into seven groups (1–7) and three columns (A, B, C). The columns present each level of Sfards’ theory of reification (e.g., task C2 represents part-whole subconstruct of fractions in reification level of fractions’ understanding) [28].

The first three triples of tasks represent on part-whole subconstruct of fractions. The measure subconstruct of the fraction is tested by the fourth triple of tasks. The other three triples of tasks are focused on equivalence, comparison, and addition of fractions. Solving some tasks required only using algorithm procedure, difficulted tasks required the application of both conceptual and procedural understanding of the concept [28].

The test consisted of 21 tasks in seven triads reflecting understanding at each stage of Sfard’s scheme in the two fraction subconstructs and the three operations, respectively.

Pantziara and Philippou developed and validated a test measuring level of mathematical successes in solving fractions task (MPF). Their test was focused part-whole and measure subconstruct, along with Sfard’s framework [28]. The choice of these two subconstructs was based on previous research [29,30].

Students’ solutions were encoded in binary form (0 if the solution was wrong, 1 if the solution was correct).

It was used the Rasch model to analyze the fraction test. We have modified some of the tasks, because of using a test in our terms. We encoded students’ solutions in binary form (0 for the wrong solution, 1 for the correct solution). We excluded the B6 item—to compare a pair of fractions with two ways. This reasoning is relatively unusual in our circumstances [28]. We also excluded the C1 item at Pantziara. We evaluated the data by the IRT model.

**IRT model**

The IRT and MIRT models were selected in this research as the main method for several reasons. First, one advantage of IRT models over linear factor analytic methods is that information from examinee response patterns is analyzed as opposed to the more limited information from correlation matrices. Second, nonlinear models such as IRT models may better reflect the relationship between item performance and the latent ability. Third, the multidimensional IRT models have been used to assess dimensionality of tests in which items reflect different skills, knowledge, or cognitive processes.

## 3. Results

**Figure 1:**
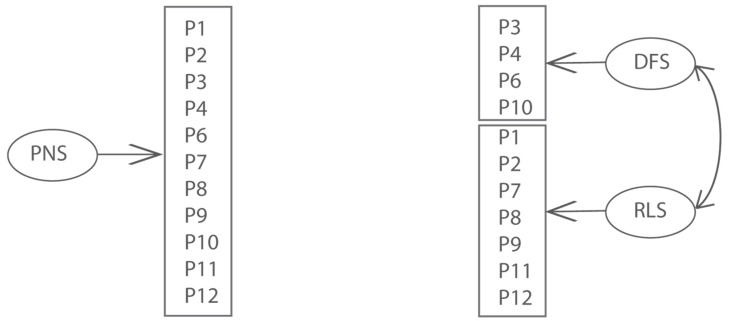
Two alternative models for PNS item responses.

The data file has been validated for using two models for the PNS scale through IRT. The first model (M3) was the unidimensional graded response model and the second (M4) was the correlated traits model (between-item multidimensional IRT model—MIRT, Figure 1).

We estimated item parameters according to the correlated traits model, using the ML estimator.

Because unidimensional (M3) and correlated traits model (M4) are nested, their relative goodness of fit can be compared using the likelihood ratio test. The resulting value (∆χ^2^(1) =23.89, *p* < 0.001) is highly significant. Therefore, the unidimensional model fits significantly better than the correlated traits model. However, comparing the unidimensional model to the correlated traits model, using the AIC and BIC reveals that the correlated traits model may be preferable: M3: AIC = 5559.3, BIC = 5763.0; M4: AIC = 5537.4, BIC = 5744.3. Therefore, in agreement with previous studies [2,19], the PNS scale measures two distinct but related components of the need for structure construct.

**DIF**

Before evaluating the properties of the eleven PNS items using the correlated traits model for two groups of respondents, DIF analyses were conducted to investigate the equivalence of item functioning for these groups. For detecting DIF in groups UPV and PEP we used SIBTEST (Simultaneous Item Bias Test), which is similar to the Mantel–Haenszel approach for detecting DIF. Based on the results of SIBTEST, we note that none of the items have a DIF.

**Estimates of parameters**

In Table 1, we present parameter estimates for the correlated traits model and the goodness of item fits statistics. A nonsignificant value of S-$\chi^{2}$ statistics for all items suggest sufficient goodness of fit for all items.

We compared two models. The first model (M3) was a unidimensional graded response model and the second (M4) was a correlated traits model (between-item multidimensional IRT model—MIRT).

**Figure 2:**
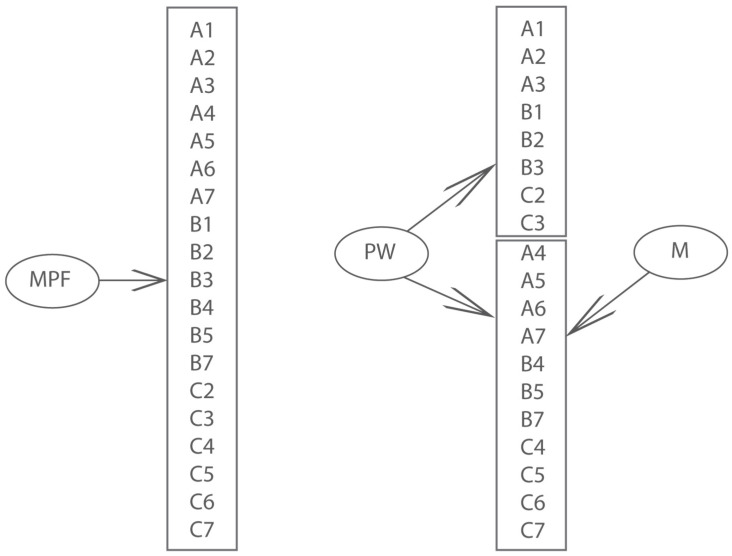
Two alternative models for Fraction test items.

The data file has been validated for using two models for the fraction test. The first (M1) was the unidimensional model (Rasch and 2PL). The second model (M2) was a bifactor model, in which the items indicate two factors—the general part-whole (PW) and the specific measure (M). The general factor corresponds to the common variance shared by all items. The specific factor is the “residual“ dimension correlated with the general factor, accounting for any remaining common variance specific to measure(Figure 2).

Because the unidimensional Rasch model (M1) and bifactor model (M2) are nested, their relative goodness of fit can be compared using the likelihood ratio test. The resulting value (∆χ^2^ (29) = 95.89, *p* < *0*.001) is highly significant. Therefore, the unidimensional model fits significantly better than the bifactor model.

The goodness of fit of the Rasch model was evaluated using M2 statistics and its associated RMSEA values. The M2 chi-square statistics (χ^2^ (170) = 245.45; χ^2^/df = 1.44) would suggest rejection of the model, but RMSEA value based on this statistic (RMSEA = 0.05) suggests a reasonably fair approximate fit.

**DIF**

DIF indicates a difference in item performance between two comparable groups of examinees, that is, the groups that are matched to the construct being measured by test [31]. We use Mantel—Haenszel approach (MH) for DIF detection [32]. *p*-values for all test items were greater than 0.05, so we note that none of the items have a DIF. Both groups (PEP, UPV) are equivalents.

**Estimates of parameters**

Table 2 presents the percentage success rate for resolution of the tasks of the fractions test (%), factor loading, difficulty, and chi-square tests for the MPF scale. Location parameters (difficulty) indicate that items were spread over the continuum of the scale from −2.24 to 3.79. Thirteen items were on the left-hand side of the scale (below the mean respondent score) and the other six items were on the right-hand side (above the mean). Chi-square tests showed that all items except for the items A1, A6 and C7 were nonsignificant (*p* > 0.05), which suggests that all other items were well fitted by the Rasch model.

**Person score**

Because this model is only one trait, we produce person scores using the EAP estimator. The EAP scores estimated using the Rasch model range from −2.12 to 2.72 (mean = 0.000, SD = 0.89) for MPF. The standard deviations are much smaller than SD = 1 assumed for the latent trait.

**Reliability**

To compute the empirical reliability of the EAP scores we use the squared standard errors of observed scores. For the MPF scale, the empirical reliability is 0.82. According to [33], this is a sufficient value.

**Analysis of the dependence of fractions knowledge and PNS**

The research aimed to find the relationships between mathematical knowledge and the cognitive-individual variable. We assume that there is a negative correlation between the personal need for structure and the success of the fraction test. We have used the IRT model for statistical evaluation. We estimated the values of the latent variables MPF (mathematical performance in the fractions), DFS (desire for structure) the RLS (reaction to lack of structure).

We have found a statistically significant correlation (negative) between MPF scores and DFS scores (r_S = −0.24, *p* = 0.002) and between MPF scores and RLS scores (r = −0.23, *p* = 0.004). Students with a low Need for Structure or signs of frustration tolerance, tolerance for uncertainty, and easy use of cognitive structures, demonstrate better results in the mathematical test.

## 4. Discussion

Research is an original example of the fusion of two disciplines (mathematics and psychology) through the general psychological concept of the need for structure. The main subjects are mathematics teachers, resp. future teachers of mathematics at the primary level, who can play a significant role in the effective grasp of mathematics issues by pupils.

We agree with the statement that the solutions of fraction tasks present a significant variable. It is influenced by the need for structure [18]. The results of our research agreement with the results of further research, e.g., [34]. According to [35], persons with a high need for structure are significantly less creative, but significantly more use the so-called algorithmic orientation in solving complex situations, which has also been proven in our research. The fraction test was not focused on the use of algorithms, it required different interpretations of fractions. The analysis of student solutions showed several problems with understanding fractions at the informal level. We found several misconceptions about fractions: determination of a part of the whole on the geometric model of the fraction, identification of equal parts of the whole; expression of a part of the whole in the form of a fraction; notation of a fraction as numbers in the form: numerator, fractional line, denominator, the relationship between the fraction and its corresponding decimal number; location of the number on the number axis; arrangement and comparison of fractions (confusion with the arrangement of natural numbers); identification of the unit line and its distribution on the numerical axis.

These results correspond to the research of [36]. Students often perceive a fraction as an object of arithmetic operations, and so it is a kind of ordered pair of numbers for them. Algorithms for working with fractions are mastered, but they cannot use the language of fractions in modeling real situations.

We prove that the success of solutions to fraction test depends inversely on the need for structure, meaning that the higher the total score of the PNS and its subfactors DFS and RLS, the lower the success rate in solving fraction test. It turned out that the students who achieved the weakest results in the test prefer a simpler structure in life and have a stronger negative experience in situations that they perceive as insufficiently structured.

The fraction test can present an ambiguous situation for students with a high need for structure. This fact can reduce their success in solving [37,38]. Therefore, it is necessary to use different models and subconstructs of fractions when creating fractions concepts. This will allow students to better classify their knowledge into structures and better understand the issue.

We can conclude those fraction problems and their solutions prove to be an important variable. It turns out that the success of their solution may be related (among other things) to the need for structure. The impact of the personal needs for structure on mathematical success is still a weakly explored area of research, but it can significantly affect the results that students achieve in mathematics.

## 5. Conclusions

In the research, we applied knowledge from personality psychology to the theory of teaching mathematics.

We have placed the personal need for structure and solving tasks with fractions at the center of interest. We applicated the fraction test in Slovak language and Slovak conditions. The fractions test can be considered a sufficiently reliable tool and with small modifications, we can use it in the conditions of Slovak schools. We analyzed student solutions to problems focused on fractions and we found statistically significant dependencies between the cognitive-personality variable personal need for structure and the success of solving problems with fractions.

The analysis of student solutions identified several persistent problems with understanding fractions at the informal level. The biggest misconceptions manifested themselves in several areas when working with fractions, examples of which are as follows: determining a part of a whole on a geometric model of a fraction; expression of a part of a whole in the form of a fraction; notation of the fraction as numbers in the form: numerator, fractional line, denominator, the meaning of the numerator and denominator of the fraction for the expression of the number of determining parts and the number of all parts into which the whole was divided; location of the number on the number axis; arrangement and comparison of fractions with a tendency to proceed from the natural arrangement of natural numbers.

The described misconceptions can be related to the specific meaning and concept of fractions in comparison with natural numbers. Fractions represent a more complex structure than natural numbers. Working with fractions can be an ambiguous situation for a student and cause more uncertainty.

It has been shown that the success of the fraction test negative correlates with the need for structure. This means that the higher the overall score scale of PNS, as well as its subfactors, the lower the success in solving problems with fractions.

We see the further direction of research in the analysis of the need for structure in other areas of mathematics, including geometry, as well as the creation of intervention mathematical programs that would consider PNS.

## Figures and Tables

**Table 1:** Factor loading, parameter estimates, the goodness of item fit statistics for correlated traits model.

Items	Factor Loading	Item Parameter Estimates						Item Fit Statistics		
		b2	b3	b4	b5	b6	a	$S-\chi^{2}$	df	*p*
Factor 1: Desire for Structure (DFS)										
P3	0.73	−2.70	−2.34	−1.32	−0.24	0.69	1.07	49.64	37	0.080
P4	0.62	−3.19	−2.45	−1.69	−0.49	0.68	0.79	55.01	40	0.057
P6 (reversed)	0.57	−2.26	−1.28	−0.24	0.96	2.13	0.70	59.86	51	0.185
P10	0.69	−2.52	−1.79	−0.40	0.73	1.92	0.96	45.56	37	0.158
Factor 2: Response to Lack of Structure (RLS)										
P1	0.53	−4.06	−3.46	−2.09	−0.06	1.49	0.62	34.20	33	0.410
P2 (reversed)	0.48	−2.90	−1.23	−0.40	1.03	2.22	0.55	63.10	51	0.119
P7	0.52	−3.35	−2.23	−0.86	0.86	2.63	0.60	46.17	38	0.170
P8	0.70	−2.74	−1.82	−0.96	−0.10	0.59	0.98	45.51	41	0.290
P9	0.61	−2.65	−1.28	−0.38	0.85	1.67	0.76	49.83	49	0.440
P11 (reversed)	0.50	−2.39	−0.80	0.60	1.84	3.11	0.58	43.00	46	0.599
P12	0.70	−2.49	−1.43	−0.60	0.47	1.51	0.97	50.96	41	0.137

PNS scale parameter estimates using a correlated trait model (factor loading; a—item discrimination; $b_{j}$—category thresholds; S-$\chi^{2}$—item-fit statistic).

**Table 2:** The percentage success rate for resolution of the tasks of the fractions test, factor loading, item difficulty, and goodness of item fit for the Rasch model.

Task	%	Factor Loading	Difficulty (SE)	$\chi^{2}(160)$	*p*
A1	75.3	0.22	−1.15 (0.19)	306.50	0.000
A2	84.6	0.81	−1.71 (0.23)	81.20	1.000
A3	80.3	0.73	−1.43 (0.21)	111.10	0.999
A4	70.4	0.78	−0.90 (0.18)	103.84	1.000
A5	90.7	0.59	−2.24 (0.29)	103.57	1.000
A6	71.6	0.28	−0.96 (0.18)	189.32	0.056
A7	85.8	0.47	−1.80 (0.24)	132.90	0.942
B1	63.6	0.79	−0.59 (0.17)	103.08	1.000
B2	74.1	0.40	−1.09 (0.19)	157.85	0.533
B3	77.2	0.70	−1.25 (0.20)	114.99	0.997
B4	61.1	0.77	−0.49 (0.17)	111.78	0.999
B5	79.6	0.25	−1.39 (0.21)	288.30	0.000
B7	74.7	0.48	−1.12 (0.19)	139.64	0.876
C2	37.0	0.68	0.53 (0.17)	177.71	0.160
C3	32.1	0.70	0.75 (0.18)	135.15	0.924
C4	25.3	0.65	1.10 (0.20)	114.03	0.998
C5	5.6	0.69	2.79 (0.35)	106.67	1.000
C6	8.0	0.56	2.43 (0.31)	286.29	0.000
C7	1.9	0.72	3.79 (0.55)	73.15	1.000

## Data Availability

No reported data.
